# Patient and public involvement and engagement with underserved communities in dementia research: Reporting on a partnership to co‐design a website for postdiagnostic dementia support

**DOI:** 10.1111/hex.13992

**Published:** 2024-02-20

**Authors:** Claudio Di Lorito, Sarah Griffiths, Marie Poole, Chandrika Kaviraj, Martin Robertson, Neil Cutler, Jane Wilcock

**Affiliations:** ^1^ Research Department of Primary Care and Population Health Centre for Ageing Population Studies University College London London UK; ^2^ Faculty of Medical Sciences, Population Health Sciences Institute Newcastle University Newcastle upon Tyne UK

**Keywords:** co‐design, dementia, partnership in research, PPIE

## Abstract

**Introduction:**

Despite the advancements in Patient and Public Involvement and Engagement (PPIE), the voices of traditionally underserved groups are still poorly reflected in dementia research. This study aimed to report on a PPIE partnership between academics and members of the public from underserved communities to co‐design Forward with Dementia—Social Care, a resource and information website supporting people receiving a dementia diagnosis.

**Methods:**

The PPIE partnership was set up in four stages: 1–identifying communities that have been under‐represented from PPIE in dementia research; 2—recruiting PPIE partners from these communities; 3—supporting PPIE partners to become confident to undertake their research roles and 4—undertaking research co‐design activities in an equitable fashion.

**Results:**

To address under‐representation from PPIE in dementia research we recruited seven PPIE partners from Black, Asian and other minority ethnic groups; lesbian, gay, bisexual, transgender, queer+ communities; remote/rural area; religious minorities and partners living with rare forms of dementia. The partners met regularly throughout the project to oversee new sections for the study website, refine existing content and promote the website within their communities.

**Conclusion:**

Strategies can be used to successfully recruit and involve PPIE partners from underserved communities in co‐design activities. These include networking with community leaders, developing terms of reference, setting out ‘rules of engagement’, and investing adequate resources and time for accessible and equitable involvement. These efforts facilitate the co‐design of research outputs that reflect the diversity and complexity of UK contemporary society.

**Patient or Public Contribution:**

This study received support from seven members of the public with lived experience of dementia from communities that have been traditionally underserved in dementia research. These seven members of the public undertook the role of partners in the study. They all equally contributed to the study design, recruitment of participants, development and revision of topic guides for the interviews and development of the website. Three of these partners were also co‐authors of this paper. On top of the activities shared with the other partners, they contributed to write independently of the academic team the section in this paper titled ‘Partners' experiences, benefits and challenges of the partnership’. Further, they provided input in other sections of the paper on a par with the other (academic) co‐authors.

## 1 INTRODUCTION

Patient and Public Involvement and Engagement (PPIE) in research is engagement with and inclusion of people who have lived experience of a phenomenon under investigation. In PPIE, research is conducted ‘with' or ‘by’ patients and the public, compared to traditional paradigms of research, where studies are carried out ‘to’, ‘about’ and ‘for’ them.^1^ PPIE debunks the myth of hierarchical knowledge, passed by academic experts to lay members.^2^, ^3^ In PPIE, members of the public are valued ‘experts by experience’, who complement and supplement knowledge contributed by academics, the ‘experts by training’.^4^ The rationale is that by combining the unique lived experience and perspective of PPIE contributors and the technical expertise of academics, research (and subsequent service design/implementation) addresses the needs and priorities of the public.

PPIE can be visualised on a continuum.^5^ The one end features lesser involvement activities for patients and the public, such as advisory roles.^5^ A typical example of this type of PPIE is a consultation to read research proposals to ensure they match the research needs of the public. At the opposite end of the spectrum is user‐led research, in which service users decide on the issues and questions to be investigated, design and carry out the research. In the middle of the continuum lie those types of PPIE that view knowledge as co‐constructed through shared contribution in the research of academic and lay perspectives.

Research partnerships (often referred to as co‐research) is the equal share of power between experts‐by‐training and experts‐by‐experience in all stages of research, from its inception to dissemination of findings.^6^ Authentic partnerships may generate a number of benefits, including improved relevance, usefulness, quality and validity of the research; greater trust within communities where research is conducted; increased empowerment of those who partner in research, and opportunities for typically under‐represented communities.^7^

Within dementia research in the United Kingdom, PPIE has gained momentum in the last decade, fuelled by the request of funders to embed in research grant bids a description of meaningful PPIE activities and monitoring systems to ensure these are undertaken equitably.^8^ While progress in PPIE in dementia is evident, challenges presenting when working with people with cognitive deterioration (especially remotely, following the COVID‐19 pandemic) led to their involvement in research mostly in advisory roles.^9^, ^10^

There has also been a challenge to represent the voices of traditionally underserved groups within dementia research,^11^, ^12^ which has resulted in neglecting the distinct needs of such communities in service design, provision and improvement.^13^ Some unanswered community‐relevant research questions include how to address barriers of South Asian communities to seek support and access dementia services,^14^ how to ensure that dementia information is accessible to deafblind users,^15^ or how to engender culture‐relevant principle of person‐centred care to support lesbian, gay, bisexual, transgender, queer+ (LGBTQ+) people with dementia and their partners/families of choice.^16^ Through an ethical and human rights standpoint, there is a need to fill in this gap, by embracing collaborative approaches and appreciating the benefits of undertaking PPIE with traditionally underserved communities in dementia research.

This methodological study aimed to contribute to the advancement of PPIE of communities that have been traditionally under‐represented in dementia research to inform future practice for researchers, PPIE contributors and funders. The objective was to report on the activities, practicalities, experiences, benefits, and challenges of a partnership between academics and members of the public from underserved communities.

## 2 MATERIALS AND METHODS

### 2.1 Design

This was a methodological study, as per the definition reported in Mbuagbaw et al.: ‘A study that describes or analyzes methods (design, conduct, analysis or reporting) … to inform methodological advances…, understand current practices, and help to identify the need for guidance and gaps in methodological or reporting quality’.^17^

### 2.2 Setting

The research partnership was embedded in Forward with Dementia (FwD)‐Social Care, a 12‐month project funded by the National Institute for Health and Care Research (NIHR) School of Social Care Research. The project aimed to develop web‐based resources for social care practitioners to best support clients living with dementia and family carers.

The project was based on co‐design, as per operational definition by Slattery et al.^18^: ‘the meaningful involvement of research users … in a research project, where ‘meaningful involvement’ is … participation in an explicitly described, defined and auditable role or task necessary to the planning and/or conduct of health research’. ‘Research users’ are ‘consumers, clinicians or other people or groups (other than researchers themselves) that have an interest in the results of health research’.^18^

In line with principles of co‐research and to ensure that the voices and views of underserved communities were reflected in the final resource, a PPIE partnership was developed for the project.

### 2.3 Sample and recruitment

Recruitment of PPIE partners occurred through first learning about issues of underrepresentation in research. We did this by undertaking a search of the existing literature on Google Scholar using terms: ‘underserved’, ‘groups’, ‘research’, ‘UK’, to identify reviews of the literature identifying communities that have traditionally been excluded in research, either as research participants or, where reported, as public contributors. Once we identified these communities, partners were recruited through personal contacts and the network of the study team and through liaison with key leaders of organisations supporting the communities.

### 2.4 Data collection

Academic leads (S. G. and C. D. L.) recorded in an activity log all activities of the partnership, including supporting recruitment of research participants, interpretation of findings, project co‐design oversight, promoting the resource and taking part in dissemination. They also compiled individual notes and reflections about the experiences, benefits and challenges of being involved in the partnership, which the PPIE partners also did.

### 2.5 Data analysis

Data on the partnership's activities were analysed through deductive thematic analysis.^19^ Themes were set a priori using a previously published template^6^ based on the NIHR Research Design Service (RDS) guidance https://www.rdsresources.org.uk/. The template identifies four stages of the research cycle: ‘Thinking and Learning’, ‘Planning and Recruiting’, ‘Preparing’ and ‘Doing’, which were used as pre‐defined themes. Data from the academic leads' notes were coded by the first author (C. D. L.) into one of these four themes. The analysis was presented to the wider team for validation/refinement. Data are presented in ‘Results’ through the four themes. All academic and PPIE partners' notes on experiences, benefits and challenges were gathered at the end of the partnership by CDL, merged into a cohesive narrative and reported for PPIE partners and academic partners verbatim (i.e., without any alteration) as joint statements (by group).

## 3 RESULTS

### 3.1 Partnership's activities

#### 3.1.1 Stage 1: Thinking and learning

Through the Google Scholar search, we identified a review of literature by the NIHR Innovation Observatory which found three umbrella factors associated with limited inclusion in research and identified the following underserved groups^20^:

1. *Demographic factors*: Black, Asian and ethnic minorities, LGBTQ+.
2. *Social and economic factors*: people with lower socioeconomic status, people living in remote areas, religious minorities, people with unequal access to health and/or social care in their locality/geographic location.
3. *Health status*: people with dementia are generally less involved in research than key informants, particularly people with rare forms of dementia, early onset dementia, people with comorbid conditions, those with visual and/or hearing impairments and those who live alone.

The review^20^ further identified barriers to inclusion in research. Although different issues affected different groups to varying degrees, common barriers included:

1. Barriers relating to cognitive impairment and capacity.
2. Barriers caused by physical disability such as mobility issues.
3. Feeling unqualified to take part (e.g., due to lack of education, training, confidence, competency).
4. Lack of effective incentives for participation.
5. Lack of interest/trust in research.
6. Negative financial impact.
7. A person lacking insight into their diagnosis.
8. Requirement for carer cover.
9. Cultural barriers such as gatekeeping by community leaders.
10. Pragmatic barriers such as time and the necessity to keep to a routine.
11. Language/literacy barriers.
12. Change in circumstance (e.g., deteriorated health).
13. The requirement for involvement is perceived as ‘too much’.

The identification of these groups and common barriers relating to involvement in research‐informed recruitment of partners (stage 2).

#### 3.1.2 Stage 2: Planning and recruiting

The main question the team reflected on at this stage was ‘how’ to ensure optimal outreach and minimise the barriers to the participation of partners from underserved communities. From previous experience of the research team, a key barrier to recruiting members from underserved communities includes a lack of access to key contacts that could act as a bridge between academia and the ‘real world’. Traditionally in PPIE, public contributors are recruited through snowball sampling, that is, word of mouth from peers already doing PPIE. The issue at play with under‐represented communities is that a lack of representation in existing research PPIE groups becomes cyclical and prevents the potential to recruit diverse PPIE members through snowball sampling.

We therefore considered alternative recruitment strategies. We liaised with community organisations, so that we could build relationships with the key people (e.g., leaders) that could support access to the most appropriate public contributors. In doing so, we also carefully considered the risk of ‘gatekeeping’ that leaders can present to accessing community members.

The team eventually agreed that the most time‐efficient and pragmatic way (for a 12‐month project) to identify partners was through personal contacts and the network of the project team. In this respect, it was helpful to have an experienced and reputable team with established contacts in diverse communities. Diversity within the academic team members was also instrumental to the success of partners' recruitment. The fact that researchers in the team were members of some of the identified underserved groups to be involved in the partnership and could use their personal contacts with organisations to broker the project meant easier access to particular groups. This enabled the team to recruit seven partners from the identified communities, all of whom had lived experience of dementia, either as a person with the diagnosis, or a (present or former) family carer (Table 1 for partners' characteristics).

**Table 1:** Partners' characteristics.

Partner's pseudonym	Gender	Age group	The lived experience of dementia	Group/community	Locality
George	M	60–75	Former carer	LGBTQ+	Northeast England
Ryan	M	60–75	Person living with dementia	Rare form of dementia (posterior cortical atrophy)/visually impaired	Scotland
Rupinder	F	30–40	Current carer	South Asian	London
Robert	M	30–40	Current carer	Mixed (Asian, Black), Muslim	East Midlands of England
Maggie	F	60–75	Former carer	Rural community	Southwest England
Anthony	M	60–75	Person living with dementia	Young‐onset dementia	Northeast England
Marianne	F	75–90	Person living with dementia	Living independently (no carer)	Northeast England

Abbreviations: F, female; LGBTQ+, lesbian, gay, bisexual, transgender, queer+; M, male.

After recruitment, some pragmatic considerations were made when planning the partnership. As per the protocol, the project (and all related PPIE activities) was to be carried out remotely via Zoom. The team was aware of the potential difficulties for people living with dementia to contribute remotely in a meaningful way. For example, remote meetings would require the ability to use information technology, which could pose a barrier to access for people with cognitive impairment and/or living alone. Strategies were therefore deployed to minimise the risk of tokenistic involvement (see Box 1).

Box 1 **Strategies for supporting engagement in remote meetings.**

- To ease anxieties about contributing remotely, facilitators spent time building relationships with contributors before group sessions, demonstrating respect that people have rich backgrounds and life experiences. The concept of co‐design was explained, and it was reinforced that everyone has something valuable to contribute to the process, thus dispelling fears about contributing e.g., ‘I don't know anything about overseeing a study.’
- Preferences regarding spoken and written communication, and any worries about communicating online were noted and steps put in place to address them. Opportunities were provided for one to one meetings via Zoom or on the phone in between or instead of meetings where preferred. Information needs were established e.g., some contributors required hard copy, large print documents sent in the post prior to meetings. Facilitators completed University expense forms for the group, to reduce contributor burden.
- Potential technical difficulties were identified, and individual help provided. For instance, if contributors had difficulty ‘getting into’ zoom meetings, a facilitator would be available on the phone to talk them through the process and offer reassurance.
- Facilitators were mindful that without the usual non‐verbal cues available in face‐to‐face meetings, people living with dementia in particular may find it difficult to indicate they had something to say and ‘take the floor.’ Particular attention was given to ensuring that everyone was given space to contribute if they wished to do so and working to avoid meetings being dominated by those without dementia. Everyone practised and was able to use the ‘raise hand’ zoom function when they wished to speak.
- To counteract potential difficulties with language comprehension, hearing, and/or fatigue, facilitators used linguistic strategies to support inclusion, such as asking ‘Was that clear?’ ‘Shall we go over that again?’ or ‘Let me know if you need a break.’
- Breaks were built into the 90‐minute meetings and the number of items on the agenda limited to one or two, to prevent people becoming overwhelmed or fatigued.
- It was acknowledged that often ideas and suggestions might occur to contributors after the conversation had moved on, but that it was OK to voice them at any stage or use the ‘chat’ facility if preferred. It was also emphasised that contributors could email the facilitators outside of meetings if ideas came to them later.

However, it was also recognised that remote working presented some important advantages. For example, it enabled recruitment/involvement of partners from a wide geographical area, and those with reduced mobility, sensory difficulties and other health issues, who could partake in partnership activities without the need to travel/leave home, and who could participate in discussions without the requirement to be present in person or to be seen on video (by turning the camera off)—anonymity being an important element for some people.

Part of planning an equitable and honest partnership also required open discussion about the nature of the proposed involvement. Some of the important questions to be addressed included what the purpose of the partnership was, what tasks the partners would be asked to take part in, what their time commitment would be, and information about financial remuneration for PPIE activities as per NIHR guidance. It was agreed that developing a Terms of Reference (ToR) document would clarify these issues and manage expectations effectively.

The resulting ToR document (Supporting Information S1: Appendix 1), based on a template used by SG in a previous project and co‐produced with two people with lived experience of dementia, outlined the background to the project, the aims and role of the partnership, membership, accountability and contact information. It was signed by the two academic PPIE leads and passed to the partners, who were invited to familiarise themselves with it, discuss whether they wanted any amendments to be made, and sign off the document.

Another strategy to manage partners' expectations and guard against any future disappointment was being honest and realistic about the likely impacts of the research. While not part of the ToR, the team ensured that this conversation was held at the beginning of the partnership and revisited throughout.

#### 3.1.3 Stage 3: Preparing

Once the seven partners had been recruited into the study, an initial two‐hour introductory meeting was held. The purpose of the meeting was to introduce the project and its key stages, as well as to establish roles. The session started with everyone introducing themselves with a fun fact.

Most of the session was dedicated to a discussion on how the partnership would run. The ToR document was used as a starting point to establish and develop consensus on ground rules, expectations, activities, payments and to answer any questions that the partners might have. In terms of the PPIE activities pertaining to this partnership, a discussion was held around partners' expectations, aspirations, skills and confidence levels to undertake research tasks, in line with the principle of ‘to each their own’. This was also instrumental in identifying partners' needs to become confident to undertake their research roles by offering, where needed, training to develop and enhance skills.

Following the discussion, it was agreed that partners would all equally contribute to:

1. Overseeing the new social care sections of the dementia guide.
2. Refining the existing sections of FWD with a focus on representation.
3. Disseminating and promoting the project and guide with their respective communities.

These activities would be undertaken in the context of three 90‐minute remote discussion meetings. If the partners could not attend the group meetings, they would be offered follow up individual meetings with one of the academic PPIE co‐leads. It was agreed that the partners would only work on project material and research activities within the group meetings, and not independently in their own time, to ensure that they could be financially reimbursed for their time.

#### 3.1.4 Stage 4: Doing

As agreed with partners, three 90‐minute remote discussion meetings were held. In the meetings, the partners co‐designed prototypes of the web resource by sharing ideas and suggestions on content and format. Following each session, the academic team and website design agency would integrate their input into a new iteration of the resource, which was presented to partners in the subsequent session. Partners were provided with a list of the changes made by the website design agency following the previous session and some queries guiding co‐design in the following session.

A number of iterative actions were also undertaken to ensure effective and equitable continued involvement of PPIE partners, including:

1. Regular discussions to revisit what was expected of partners at each stage of the research process and what they could expect from the academic team.
2. Ongoing support to equip and empower partners to engage meaningfully, including strategies to support them in remote meetings (Box 1).
3. Feedback from researchers about how the partners' contributions were directly shaping the research and feedback from the partners about their ongoing experiences so that changes could be made swiftly where needed.
4. Opportunities for the research team and partners to get to know each other more informally, to break down barriers of ‘them and us’, and foster empathy and equality.
5. Discussions and development of plans for continuing partnership (beyond the project).

All research activities and iterative actions were underpinned by the academic team abiding by the following key principles:

1. Ensuring interactions were based on respect, humanity and empathy.
2. Ensuring power was distributed equitably and fairly throughout the partnership process (e.g., promoting equal input in partners' discussions of those who were reluctant to speak up publicly, through the use of chats and separate individual meetings).
3. Ensuring equitable access for partners (e.g., meetings do not impede partners' work, care, religious or health commitments).
4. Addressing power relations by not using titles, ensuring that partners were not outnumbered by research team members, agreeing ground rules such as not interrupting and offering criticism constructively and respecting all views.

#### 3.1.5 PPIE partners' experiences, benefits and challenges of the partnership

Our overall experience was very worthwhile. This project was the most practical health research that we have been involved with. From the very first meeting, we were able to see the final outcome goals. Many research projects hint at real world outcomes but you are not physically part of them. We felt that this is what true public engagement is about—collaboration and partnership. We saw the results of the ideas and feedback we provided in a website, which will be used by a range of people. We are living dementia day‐to‐day, and this journey can be very downbeat. Being part of the group helped. Even for one or two hours a day, it was better than nothing. And channelling our experiences into a project which can help people like us but also professionals was empowering.

There were many benefits that we gained from being part of the group. Each one of us highlighted points and shared experiences that the others had never thought of. We were all able to speak and taken as equals. The academic team did not name themselves higher than us. And they devolved power to us as well. They came in with an open mind, not knowing what the end product would be. They just heard what we had to say. We learned so much: from campaigning and running groups through to conference participation, web design and accessibility. Through our group, we also became friends with and supporters of each other. We have arranged to visit each other because we live so far away. That's a very positive thing. And we are also going to write our own papers together.

We felt that there was a good cross section of people in the partnership. In fact, it was the most diverse group of people we have worked with. However, only one of us actually has dementia. This is a recurring problem with much research. We feel that part of the problem is that taking part in research is not upsetting, but it is not positive or uplifting either. And for people living with dementia, it is sometimes difficult to hear about similar difficult experiences. This is especially true in some groups, such as people with early onset dementia, who are seven times more likely to end their lives than anyone else. Some of us have actually been ostracised by other groups for discussing for example mental health. While we cannot excuse that, we can certainly empathise and excuse people for not liking it.

Our representation could have been a bit wider also from the LGBT communities. One thing we have learned by experience is the difficulty to actually get people to sign up to PPIE, especially older LGBTQ+ people who still feel a bit scared about sharing their life stories. In this group, we tried to be as far reaching and wide looking as possible. We invited people from many LGBTQ+ groups, but they declined the invitation. We thought this was a shame because you have got a story to tell and something to add which would be really worthwhile.

Another limitation of the PPIE activities was that basic tech savviness was key to contribute. As people who are reasonably IT proficient, we were quite able to input. But we feel that there are many who would not be able to contribute.

We reflected on some practical points for future groups. There are great benefits to doing meetings face‐to‐face. Even though one member of our group keeps his eyes closed half the time, he does prefer seeing someone's face when he opens them. It feels a bit more human. Without seeing faces, you are missing out on subtle body language. So, we feel it is quite important. But of course, there are barriers to in‐person meetings too. This would make them very local because people will not be travelling far. And we are very much scattered all across the UK in this group. So, in‐person meetings would not get all the people together. Therefore, we think it is important that future research funding allow academic researchers travel costs to visit someone rurally.

Another practical pointer for future partnerships is that there should be ice‐breaking activities at the beginning of the group. In this group, we asked what our favourite song was and why. We found that little ice breakers showed our human side and helped build rapport with each other. The other practical pointer would be to set meeting dates ahead of time, ideally discussing two or three dates at the end of each meeting to make sure that people are available. Sometimes, if we leave the meeting date until late, people might have already committed to other things. So, it is about just trying to do a bit of forward planning to ensure attendance over time.

### 3.2 The academic team partners' experiences, benefits and challenges of the partnership

There was much mutual learning (reciprocity) from this experience. As researchers, we learned to appreciate the barriers in pursuing better representation of groups (e.g., all LGBTQ+ communities) that have been traditionally underserved in society and that as such are still hesitant to be involved in PPIE. This came with an appreciation of the time it takes to develop relationship and engender trust with underserved communities. It is key to acknowledge that each community have their own culture and, that without clear strategies to identify/recruit within these groups and terms of reference informing “rules of engagement”, it is unlikely that certain “hidden” group can be successfully involved in research.

Once successful recruitment occurred, we appreciated the importance of an iterative process of cultivating rapport and collaboration. For example, regular catch ups with partners were held on an individual basis by different members of the academic team. This, we felt, ensured that the partners felt valued as individuals not only in relation to steering research but as human beings, as reported directly by our partners. It also helped to communicate clearly and regularly about the tangible outputs to be obtained from the partnership/project. The key transferable learning for other researchers is that research bids and proposals should seek to build in time and resources to undertake such activities.

In relation to project outputs, i.e., integrating partners' views into the development of the online resource, we learnt the importance of attitudes, values, and behaviours on the part of all collaborators including the academic team and website design agency. To accommodate the views and input of partners, it was key to approach the partnership activities with a flexible attitude, with skills to be able to mediate disparate often contrasting views, a mindset that is open to accept criticism and constructively build on it, and an ethos that acknowledges how valuable points can be contributed by all partners. In fact, that knowledge thrives when different expertise combines. Therefore, creating a culture that challenges traditional views on power relationship, knowledge, and science and that engenders out‐of‐the‐box thinking is key.

In terms of practicalities, much is to be learnt by the fact that our partnership was entirely undertaken remotely. This had undeniable benefits, enabling participation of partners form rural areas and/or with mobility issues, and ensuring diversity in the group. However, in terms of accessibility, holding all partnership's activities online via videocall platforms required careful considerations of ways in which to ensure that all partners could connect and participate to the online sessions. The academic team held individual sessions with partners who needed training/guidance on, for example, how to use the different functions of video calling platforms. When partners struggled to come to the sessions or interact virtually, dedicated one‐on‐one debriefing sessions were held to ensure that their views were gathered. Another practical challenge pertained to paying PPIE partners. The inevitable delays that organising payment with academic institutions’ financial department entailed needed constant chasing up and extra work for the academic team and a potential impact on the relationship with partners.

Finally, this partnership was a unique opportunity for us researchers to develop personally as human beings, with a greater appreciation of the difficulties that underserved communities experience when it comes to dementia diagnosis, support and care. We believe that having developed this further awareness and empathy has made us even more committed researchers trying to make change for the community of people with dementia’.

## 4 DISCUSSION

This study aimed to contribute to the advancement of PPIE of communities that have been traditionally underrepresented in dementia research. It reported on how a partnership between the research team and members of the public from underserved communities was developed and how oversight of co‐design occurred throughout a project developing an online dementia guide to support people living with dementia and carers to live well after a dementia diagnosis and to help people in social care who are working with them.

The study successfully recruited partners from a range of communities that have been traditionally under‐represented in dementia research, such as LGBTQ+, rare form of dementias, visually impaired, South Asian, mixed ethnicity, Muslim, rural communities, young onset dementia and those living independently (with no family carer). Despite the time constraints, partners were recruited within a short period of time. This required pre‐existing conditions. Using the team's established network with underserved communities and snowball sampling to expand on these and recruit partners was key. The success of snowball sampling in the recruitment of members of traditionally underserved populations has been reported in previous literature.^21^

Successful and timely recruitment of partners was also ensured by key groundwork, such as the scoping review of the literature and the development of ToR outlining roles and responsibilities. The importance of clear agendas and ToR has been reported in previous research.^22^ While this groundwork required initial time and resources, it was an investment that fully paid back. The literature review enabled us to identify communities to be involved, and strategies to access these communities, and the ToR ensured that expectations were realistic and fulfilled.

Another important factor that made this study successful was an investment in resources. As reported in previous research,^22^ it was crucial for the academic team to invest time to develop relationships and trust with the partners and to undertake important practical tasks to ensure that involvement was undertaken in accessible and equitable ways.

Another task to ensure equitable involvement was financial retribution for partners’ time spent in the study. Our team acknowledged from the start that it could not represent a standalone strategy to ensure the engagement and commitment of our PPIE partners. Alone, financial retribution would run the risk of developing extrinsic motivation in our partners (i.e., motivation that is solely driven by external reward), as opposed to intrinsic motivation (i.e., the inherent satisfaction/commitment to making meaningful change). This is why we adopted accompanying motivational strategies, which, based on a self‐determination theory perspective,^23^ addressed our partners' autonomy, competence and relatedness needs, promoting intrinsic motivation.

We, therefore, advocate that while PPIE should rightly involve financial retribution, it should be integrated within a range of intrinsic motivational strategies addressing PPIE contributors' needs for autonomy (e.g., through sharing power in decision‐making processes), competency (e.g., through investing in their training and development) and relatedness (e.g., through cultivating rapport beyond research activities). In line with previous research,^24^ findings from this study suggest that to be able to engage with partners effectively, it is key to devolve sufficient resources in terms of costing/staff/time, which may call for a dedicated post/person to lead on all PPIE activities and/or dedicated admin support for all PPIE budgeting/financial tasks.

This study was characterised by certain strengths and limitations. It successfully recruited and involved partners from underserved communities in dementia research and from diverse background/locations, ensuring that their views and input generated the project output, an online dementia guide that responds to the needs of an everchanging diverse society. We were able to do all this within the constraints of a short‐term project of 12 months, adding to the evidence that meaningful involvement can occur in a relatively short period of time, if adequately developed, funded, and staffed.

There were some limitations generated by the remote nature of the partnership. Only one person living with dementia was able to join our partnership activities. Although support and guidance were offered on a one‐on‐one basis, we advocate for some in‐person PPIE activities to maximise the involvement of more PPIE contributors living with the dementia. Researchers should be open to travelling to PPIE members. This is especially crucial when working with communities who have an instinctive mistrust toward academia. In the words of Litherland et al., we advocate for ‘small and informal works’,^25^ as opposed to large formal remote meetings. It should be recognised that face‐to‐face activities will inevitably increase resources to be allocated to PPIE (time for travel), raise practical/logistical issues for PPIE partners (e.g., travelling to venues), and potentially pose barriers to involvement for those who are less confident for in‐person meetings. A balance between pragmatic considerations and ethical ones will be the ideal compromise to ensure the meaningful yet sustainable involvement of PPIE members from diverse communities in dementia research.

Another limitation was the fact that partnership meetings could not be scheduled far in advance. Due to the rapid iterative nature of this project, PPIE partnership meetings were pragmatically arranged when project findings emerged and feedback was needed from the partners, which was not ideal for attendance. We would therefore advocate, if possible, to have meetings in diaries as far in advance as possible.

## 5 CONCLUSION

This study showed that undertaking meaningful partnership in dementia research with members from underserved communities is feasible, and can generate benefits for the research project, and all those involved. There are certain conditions to be met to successfully recruit and involve PPIE partners from such communities. These include networking with community leaders, developing clear ‘rules of engagement’, and investing adequate resources and time for accessible and equitable involvement of partners. These efforts represent an investment in return to ensure that future research (and outputs) better reflect the diversity and complexity of contemporary society.

## AUTHOR CONTRIBUTIONS

**Claudio Di Lorito**: Conceptualisation; investigation; writing—original draft; methodology; writing—review and editing; formal analysis; project administration; data curation. **Sarah Griffiths**: Writing—original draft; validation; writing—review and editing; formal analysis; project administration; data curation. **Marie Poole**: Validation; visualisation; writing—review and editing; writing—original draft. **Chandrika Kaviraj**: Writing—original draft; writing—review and editing; formal analysis. **Martin Robertson**: Writing—original draft; writing—review and editing; formal analysis. **Neil Cutler**: Writing—original draft; writing—review and editing; formal analysis. **Jane Wilcock**: Conceptualisation; funding acquisition; writing—original draft; methodology; validation; project administration; supervision; resources.

## CONFLICT OF INTEREST STATEMENT

The authors declare no conflict of interest.

## ETHICS STATEMENT

Forward with Dementia—Social Care received ethics approval from the UCL Research Ethics Committee on 21 June 2022, Reference: 18567/003.

## Supporting information

Supporting information.

## Data Availability

The data that support the findings of this study are available from the corresponding author upon reasonable request.
