# Supplementary material for: Patient and public involvement and engagement with underserved communities in dementia research: Reporting on a partnership to co‐design a website for postdiagnostic dementia support
Source: Health Expect. 2024 Feb 20;27(1):e13992. doi: 10.1111/hex.13992 (PMC10877991; doi:10.1111/hex.13992)
Supplement: Supplementary file 1 — Supporting information. [file HEX-27-e13992-s001.docx]

# Appendix 1. Terms of Reference (ToR) document

## Name of the group: Partnership Group

Title: Terms of reference (01/06/2022) for the Partnership group for ‘Forward with Dementia-Social Care’

This group was established by Sarah Griffiths (Senior Fellow) and Claudio Di Lorito (Research Fellow); University College London (UCL) in June 2022.

The group supports the ‘Forward with Dementia-Social Care’ project; Principal Investigator Jane Wilcock (Senior Research Fellow UCL).

This document sets out: the background to the project, the aims and role of the group, membership, accountability and contact information.

## Background to Forward with Dementia-Social Care (FWD-SC)

This study aims to support people living with dementia and carers to live well after a dementia diagnosis and to help people in social care who are working with them. People living with dementia and their carers often have negative experiences of NHS and social care services around diagnosis. They have told us there is often little support and information; one said “When I was first diagnosed, I felt lost and so overwhelmed. I didn’t know where to turn.”

Many people living with dementia use homecare services to support them living at home. For people living with dementia without family or friends, homecare and other community services provide vital regular support and social contact. Most social care workers, especially homecare workers, often work alone, lack specific dementia training and are not confident in their roles.

We will build on a study that is already providing post-diagnostic support for people living with dementia and carers through an online website and toolkit called Forward with dementia: https://www.forwardwithdementia.org/. It has been developed with, and for, people living with dementia and carers. It does not require much technical knowledge and can be used through smartphones and computer tablets. Current users are people living with dementia, carers and healthcare workers.

The Forward with Dementia -Social Care study aims to further develop the online resource for social care users and include information and guides for social care practitioners, particularly people working in homecare, to help support their clients. In order to do this, we will be carrying out interviews with a range of people with dementia, carers, and social care practitioners, to inform adaptations to the online resource. We will then hold workshops with a similar group of people to adapt the online resource based on the interview findings.

## Aim of the group:

The group’s purpose is to:

• Provide feedback and advice to researchers on the suitability of research methods

• Contribute to interpreting and applying the research findings

By drawing on individual and collective experiences, the amendments to the online toolkit will be embedded in real life experiences and contexts.

## Role of the group:

• Attend 4 x 90-minute informal (and friendly) discussion meetings. These meetings are likely to be held in June 2022, September 2022, November 2022 and March 2023, however a doodle poll will be used to identify dates/times that are convenient for members. The main format for meetings will be remote via Zoom

• Discuss ideas on how to recruit a diverse range of people to the study

• Provide feedback on interview questions and findings

• Sharing experiences of dementia and social care (where members feel comfortable to do so)

• Check the content of the updated online toolkit

• Help to share the results of the study with a wider audience

• Group members who feel comfortable to will be photographed and/or will speak on camera about the findings at the end of the study.

## Membership:

Ten people who have lived experience of dementia or of caring for someone living with dementia will be invited to join the group.

Our aim is that membership will involve a range of people reflecting the diversity of the UK population in respect to age, gender, culture, ethnicity, sexual preference, physical ability, and those living in towns, cities and rural areas.

Payment will be £25 per hour, to include any preparation.

## Accountability (based on Patient and Public Involvement / Equality, Diversity and Inclusion strategy):

The group will be led and facilitated by Sarah Griffiths and Claudio Di Lorito.

To set up an honest/meaningful partnership with partners, the facilitators will:

• Be clear about the nature of the proposed involvement – What tasks will the partners be asked to take part in? What is the time commitment? What is the purpose of it?

• Find out about partners’ skills, goals, preferences and needs (e.g., language and communication needs), so that the research role can be adapted around these

• Establish in discussion the different research roles and expertise within the partnership (“to each their own”)

• Offer support to develop partners’ skills and make them confident to undertake their roles

• Provide a point of contact for partners about their involvement in the group

• Encourage feedback and reflection on the format and content of the partnership so that it can be adapted as needed

• Be honest about the likely impacts of the research

To work effectively and equitably throughout involvement, the facilitators will:

• Have regular discussions about what is expected of partners at each stage of the research process, and what they can expect from the academic team

• Present and explain research questions, methodology, analysis and findings in a clear, jargon-free and concise manner and in an accessible format

• Ensure equitable access for partners (e.g., activities and meetings do not impede partners’ work, care, religious or health commitments)

• Send any reading or preparation to the partners at least a week prior to planned meetings, in the partners’ chosen format

• At the start of each meeting, provide a verbal summary of how the partners have influenced the research. The feedback will also be provided to partners in writing

• Address power relations during meetings by: Not using titles, giving everyone the opportunity to speak, ensuring that partners are not heavily outnumbered by research team members, agreeing ground rules such as not interrupting and offering criticism constructively

• Ensure all communication and interactions are based on respect, humanity, empathy and understanding of each other’s diverse experiences

• Circulate summary notes on the discussions and agreed actions of the meeting within two weeks of each meeting

• Ensure that the partners receive constructive and timely feedback on how their contributions have influenced and informed the study and receive summary documents, where possible

• Value partners’ feedback in data collection, analysis and dissemination of findings, as well as in study outputs

• Maintain regular contact and keep the partners updated

• Provide ongoing support to equip and empower partners to engage meaningfully

• Make sure the group review the value of its work on an ongoing basis

• Value partners’ lived experience and reward their involvement financially

• Set up plans for continuing partnership (beyond the study)

## Contact information

Sarah Griffiths: s.a.griffiths@ucl.ac.uk Tel 020 3108 6699

Claudio Di Lorito: claudio.dilorito@ucl.ac.uk Tel 0745-3717277

These terms of reference will be reviewed in September 2022, or prior to this if members request such a review.
